# Evaluation of volume responsiveness by pulse pressure variability and inferior vena cava dispensability index at different tidal volumes by mechanical ventilation

**DOI:** 10.1590/1414-431X20198827

**Published:** 2019-08-29

**Authors:** Fujuan He, Xiaoqiang Li, Suman Thapa, Chi Li, Jiawei Luo, Wenyan Dai, Jin Liu

**Affiliations:** Department of Anesthesiology, West China Hospital, Sichuan University, Wuhou District, Chengdu, Sichuan, China

**Keywords:** Pulse pressure variation, Inferior vena cava dispensability index, Volume responsiveness, Tidal volume, Mechanical ventilation, Fluid challenge

## Abstract

This study investigated the effects of tidal volume (TV) on the diagnostic value of pulse pressure variation (PPV) and the inferior vena cava dispensability index (IVC-DI) for volume responsiveness during mechanical ventilation. In patients undergoing elective surgery with mechanical ventilation, different TVs of 6, 9, and 12 mL/kg were given for two min. The left ventricular outflow tract velocity-time integral (VTI) was measured by transthoracic echocardiography. The IVC-DI was measured at sub-xyphoid transabdominal long axis. The PPV was measured via the radial artery and served as baseline. Index measurements were repeated after fluid challenge. VTI increased by more than 15% after fluid challenge, which was considered as volume responsive. Seventy-nine patients were enrolled, 38 of whom were considered positive volume responsive. Baseline data between the response group and the non-response group were similar. Receiver operating characteristic curve confirmed PPV accuracy in diagnosing an increase in volume responsiveness with increased TV. When TV was 12 mL/kg, the PPV area under the curve (AUC) was 0.93 and the threshold value was 15.5%. IVC-DI had the highest diagnostic accuracy at a TV of 9 mL/kg and an AUC of 0.79, with a threshold value of 15.3%. When TV increased to 12 mL/kg, the IVC-DI value decreased. When the TV was 9 and 12 mL/kg, PPV showed improved performance in diagnosing volume responsiveness than did IVC-DI. PPV diagnostic accuracy in mechanically ventilated patients was higher than IVC-DI. PPV accuracy in predicting volume responsiveness was increased by increasing TV.

## Introduction

Fluid resuscitation is the first and key step in the treatment of septic patients with circulatory failure (1,2). Appropriate fluid therapy can increase cardiac output and improve tissue perfusion and oxygen supply, which helps improve the condition of the patient. Hypovolemia is very common in elective surgery because of preoperative routine fasting and liquid-fasting in patients (3). Perioperative hypovolemia is closely related to the post-surgical prognosis of patients. Additionally, hypovolemia without timely treatment might lead to post-operative nausea and vomiting, prolong the length of hospital stay, and even lead to end organ failure (4). Intravenous infusion helps to maintain hemodynamic stability in critically ill and post-surgical patients. Normally, fluid infusion can increase cardiac output; however, excessive fluid load might lead to interstitial edema, and even worsen the condition of the patient (5–8).

Many studies have shown that excessive fluid intake is a common phenomenon in clinical practice, including septic shock patients and high-risk surgically anesthetized patients (9–12). Current studies have shown that only about 50% of patients that present with unstable hemodynamics show volume responsiveness, meaning that the stroke volume of patients after fluid infusion would increase, and about 50% of patients would display no volume responsiveness; thus, under such conditions, fluid infusion may cause bodily harm (13).

At present, it is believed that dynamic volume indices, which are based on cardiopulmonary interactions, such as stroke volume variation (SVV), pulse pressure variation (PPV), pleth variability index, inferior vena cava (IVC) variation, and superior vena cava (SVC) variation, offer more objective and significantly reliable guidance for fluid infusion in clinical practice (14–16). Factors affecting these indices included the tidal volume (TV), positive end-expiratory pressure (PEEP) ventilation, arrhythmia, ventricular failure, and other factors. Moreover, changes in intrathoracic pressure caused by respiratory dynamics must be sufficiently large to cause periodic changes in cardiac output. In patients undergoing mechanical ventilation, TV was considered the main factor affecting the pleura, pericardial pressure, and right ventricular afterload (17,18). Compared with PPV, transthoracic ultrasonography for IVC-dispensability index (DI) has the advantages of being non-invasive, rapid, convenient, inexpensive, and reproducible.

In this study, the effect of TV on the diagnostic value of PPV and IVC-DI for volume responsiveness was explored. We hypothesized that the diagnostic accuracy of PPV and IVC-DI could be improved by increasing the TV. In addition, the optimal TV that enabled achieving the best diagnostic value was explored. Finally, this study determined which index (PPV or IVC-DI) was more accurate in diagnosing volume responsiveness, thus providing improved evidence for optimized volume therapy in clinical practice. Our study population targeted patients receiving elective surgery mechanical ventilation. In this study, the velocity-time integral (VTI) of the left ventricular outflow tract was used as the reference method to evaluate volume responsiveness.

## Material and Methods

This trial was approved by the local Ethics Committee and registered with the Chinese Clinical Trial Registry. All patients had signed an informed consent.

### Patients

We included generally anesthetized and mechanically ventilated male and female patients, aged 18–60 years, with an American Society of Anesthesiologists (ASA) grade of 1–2. Pre-operative strict fasting and drinking prohibition was observed by all patients. The exclusion criteria were as follows: pregnancy, arrhythmia, left ventricle ejection fraction <40%, presence of heart valve disease, obvious right ventricular dysfunction, right heart failure, intracardiac shunt, peripheral artery disease or stenosis, contraindication of fluid challenge (acute coronary syndrome, cardiac shock, and evidence of capacity overload), chronic obstructive pulmonary disease, pulmonary hypertension, pulmonary embolism, increased abdominal pressure, intraperitoneal giant tumor, intra-aortic balloon pump, and a positive Allen's test.

### Methods

Three research investigators (designated as A, B, and C) were mainly responsible for different parts of the study. Two of them (A and B) were trained in transthoracic cardiac ultrasound skills. Investigator “A” was responsible for recording the imaging data of the test indicators by ultrasound and did not measure the indicators. Investigator “B” was responsible for measuring and calculating the imaging data that were recorded by investigator “A” and was unaware of the other basic information and indicators of the patient. Investigator “C” was responsible for observing and recording vital signs of the patient and other indicators during the experiment and was blinded to the ultrasonic measurement data of the patient.

An electrocardiogram monitor (Mindray, BeneView T8, China) was used to measure PPV from arterial pressure waveforms and record vital signs of the patients. Transthoracic echocardiography (Mindray, M9cv) was used to measure the VTI of the left ventricular outflow tract and the diameter of the IVC. Routine anesthesia induction was comprised of the following approach: rapid induction with 0.05–0.1 mg/kg midazolam, 1.5–2.5 mg/kg propofol, 0.3–0.5 μg/kg sufentanyl, 0.15 mg/kg cis-atracurium (0.6–1.0 mg/kg rocuronium), and inhalation of sevoflurane to maintain anesthesia after endotracheal tube adjustment according to the patient-specific situation. TV adjustment was carried out after about 10 min with a post-endotracheal tube when the hemodynamics of the patient were stable. Heart rate (HR), diastolic blood pressure, systolic blood pressure, mean arterial pressure (MAP), PPV, peak pressure (Ppeak), plateau pressure, the VTI of the left ventricular outflow tract, and IVC-DI were recorded after a 6 ml/kg TV. The tidal volume was set according to the international ideal body weight. The respiratory rate of 9 cycles/min for 2 min and the air flow at 1L/min were maintained.

**Figure 1.**
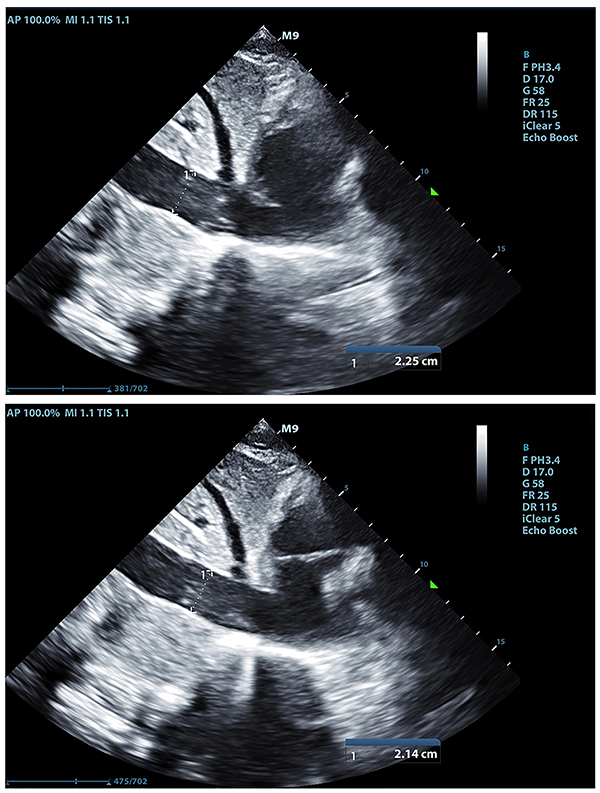
Inferior vena cava diameter at inspiration (top) and expiration (bottom) of a non-responsive group representative.

**Figure 2.**
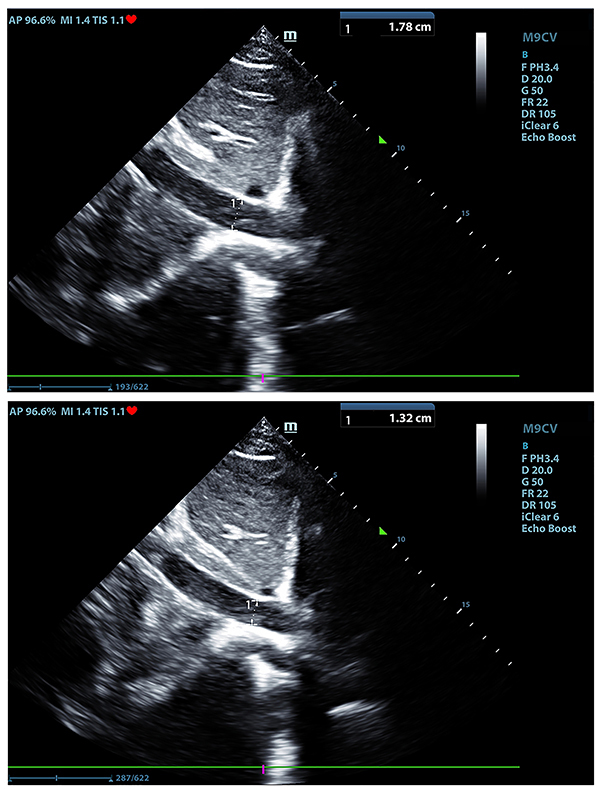
Inferior vena cava diameter at inspiration (top) and expiration (bottom) of a responsive group representative.

The velocity-time integral (VTI) of the left ventricular outflow tract was measured by transthoracic echocardiography. Blood flow of the left ventricular outflow tract was recorded by pulsed Doppler echocardiography on an apical five-chamber view. Sample volume lines were placed in the aortic annulus. The average value was taken after continuous measurement for approximately three to five times in one breathing cycle. The IVC diameter (IVC inspiration/IVC expiration) was measured at the subxiphoid transabdominal long axis, the position of the section was 2–3 cm from the distal end of the right atrial opening of the IVC. The diameter of the IVC at the end of inspiration (IVC_inspiration_) and the diameter of the IVC at the end of expiration (IVC_expiration_) were also measured. The average value was taken after three continuous measurements. The inferior vena cava diameter at inspiration and expiration of the non-responsive (NR) group is shown in Figure 1, and the inferior vena cava diameter at inspiration and expiration of the responsive (R) group is shown in Figure 2.

The IVC-DI was calculated as (IVC_inspiratory_ – IVC_expiratory_) / IVC_expiratory_ × 100%.

The TV was adjusted to 9 and 12 mL/kg and the respiratory rate was maintained at 9 cycles/min. The indices were recorded after maintenance for 2 min and the airflow was maintained at 1 L/min.

**Figure 3.**
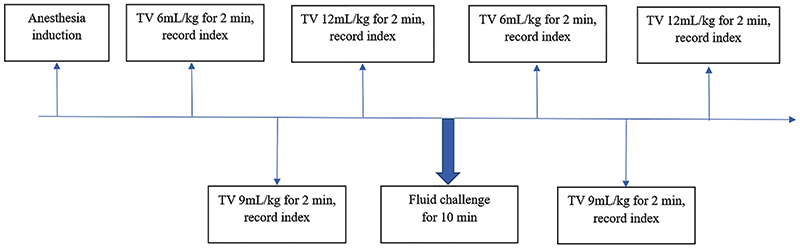
Flow chart of the study. TV: tidal volume.

*Fluid challenge.* Succinyl gelatin was infused intravenously over a 10-min period with a total infusion volume of 6 mL/kg that served to observe volume responsiveness. The tidal volumes of 6, 9, and 12 mL/kg were adjusted immediately after the fluid challenge and maintained for 2 min, and then the above indices were recorded again (Figure 3).

*Positive criteria for the fluid challenge and grouping.* Patients with an increased VTI of the left ventricular outflow tract (ΔVTI) ≥15% after the fluid challenge were enrolled in the response group (R group) and patients with a ΔVTI <15% were enrolled in the non-response group (NR group).

*Exclusion criteria during the study.* Changes of HR and MAP fluctuated by more than 20% of the baseline value or required vasoactive drug intervention; a Ppeak >30 cm H_2_O; an ultrasound image quality grading ≤3.

*Image quality grading.* Images were graded according to the following scoring system: Grade 1: no image; Grade 2: poor and unusable image; Grade 3: usable image quality; Grade 4: acceptable or good image quality; Grade 5: perfect image quality (19).

### Statistical analysis

Measurement data conforming to a normal distribution are reported as means±SD; data that did not conform to a normal distribution are reported as the median (Q25, and Q75). Enumeration data are reported as frequency (percentage). The two groups of measurement data were compared by independent samples Student's *t*-test (normal distribution). The Wilcoxon rank sum test was used for data that did not conform to a normal distribution and chi-squared test or Fisher's exact test was used to compare two groups of enumeration data. Paired sample *t*-test or a signed rank sum test was used to compare paired data. An alpha value of P<0.05 was considered to be significantly different. Receiver operating characteristic (ROC) curves of PPV and IVC-DI were plotted under different TV values. The Youden method was used to determine the threshold values of PPV and IVC-DI and to identify optimal sensitivity and specificity for diagnosing the volume responsiveness of patients. The differences in the area under each ROC curve were compared using the Delong test. SPSS version 24.0 software (USA) was used for all statistical analyses.

## Results

### Details of the recruited patients at the completion of the study

**Figure 4.**
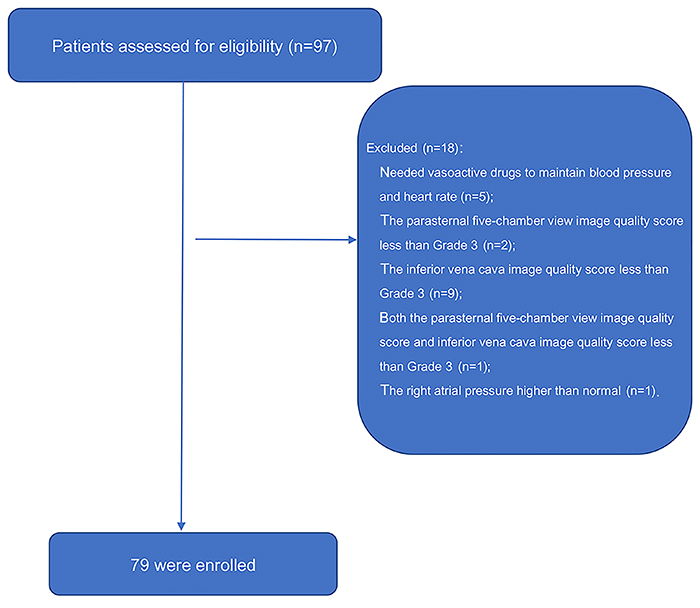
Study diagram.

From June to September 2018, 97 patients that were undergoing elective surgery in the West China Hospital of Sichuan University were included. Finally, 79 patients were selected from the recruited cohort and formally enrolled in the trial. Of these, five patients needed vasoactive drugs to maintain blood pressure and heart rate; two patients had a parasternal five-chamber view image quality score of less than Grade 3; nine patients had an inferior vena cava image quality score of less than Grade 3; and 1 patient had both an parasternal five-chamber view image quality score and inferior vena cava image quality score that was less than Grade 3. In another case, the IVC_min_ was 2.46 cm before fluid challenge was performed, and the right atrial pressure was higher than normal or volume overload was considered; thus, in this case, no fluid challenge was performed (Figure 4).

### Trial grouping and baseline comparisons

Of the 79 patients enrolled, 38 were positive for volume responsiveness. Baseline data are shown in Table 1. There was no significant difference seen in baseline data between the R group and the NR group. Baseline data of both groups were balanced and comparable.

**Table 1.** Patients' characteristics.

	Overall (n=79)	Responsive (n=38)	Non-responsive (n=41)	P value
Age (means±SD, years)	44.7±10.2	44.0±11.2	45.3±9.3	0.578
Gender (n %)				0.154
Male	29 (36.7)	17 (21.5)	12 (15.2)	
Female	50 (63.3)	21 (26.6)	29 (36.7)	
History of Hypertension (n, %)	3 (3.8)	2 (2.5)	1 (1.3)	0.512
History of Diabetes (n, %)	1 (1.3)	0 (0.0)	1 (1.3)	0.333
Thyroid dysfunction (n, %)	2 (2.6)	1 (1.3)	1 (1.3)	0.957
BMI (means±SD, kg/m^2^)	22.5±2.7	22.6±2.7	22.4±2.6	0.669
BMI≥25 kg/m^2^ (n, %)	21 (26.6)	9 (11.4)	12 (15.2)	0.575
Type of surgery (n, %)				0.266
Urologic surgery	71 (90.0)	36 (45.6)	35 (44.3)	
Breast surgery	8 (10.1)	2 (2.5)	6 (7.6)	
HR (means±SD, bpm)	79.1±11.4	81.6±11.7	76.9±10.8	0.068
MAP (means±SD, mmHg)	91.2±9.9	91.1±9.1	91.3±10.5	0.943

Continuous variables: Student's *t*-test (normal distribution) or Wilcoxon rank sum test (non-normal distribution) were used. Enumeration data: chi-squared test or Fisher's exact test were used. BMI: body mass index; HR: heart rate; MAP: mean arterial pressure.

### Comparison of baseline values between the R and NR groups

Before the fluid challenge, HR, diastolic blood pressure, systolic blood pressure, MAP, PPV, Ppeak, plateau pressure, the VTI of the left ventricular outflow tract, and IVC-DI were recorded when a TV of 6, 9, and 12 mL/kg ventilations were performed (Tables 2-4, respectively). It was found that the HR, PPV, and IVC-DI in the NR group were lower than those in the R group, and the VTI and IVC_min_ in the NR group were higher than those found in the R group.

**Table 2.** Comparison between groups in the tidal volume of 6 mL/kg group.

	Responders (n=38)		P value	Non-Responders (n=41)		P value
	Before fluid challenge	After fluid challenge		Before fluid challenge	After fluid challenge	
HR (bpm)	66.1±11.3	59.0±8.1*	<0.001	60.5±8.3^#^	56.1±6.9*	0.002
MAP (mmHg)	70.6±9.7	71.7±9.8	0.561	71.2±9.8	69.1±8.0	0.206
VTI (cm)	18.3±3.0	22.0±3.4*	<0.001	20.4±3.0^#^	20.9±3.1*	0.022
PPV (%)	10.1±3.4	5.8±2.2*	<0.001	6.7±2.6^#^	4.5±1.5*	<0.001
IVC max (cm)	1.7±0.3	2.0±0.2*	<0.001	1.8±0.3	2.0±0.3*	<0.001
IVC min (cm)	1.5±0.3	1.9±0.2*	<0.001	1.6±0.3^#^	1.9±0.3*	<0.001
IVC-DI (%)	14.1±8.2	6.0±3.6*	<0.001	9.3±7.7^#^	4.6±4.0*	<0.001

Data are reported as means±SD. Student's *t*-test (normal distribution) or Wilcoxon rank sum test (non-normal distribution) were used. *P<0.05 *vs* before fluid challenge; ^#^P<0.05 *vs* responders. HR: heart rate; MAP: mean arterial pressure: VTI: velocity-time integral; PPV: pulse pressure variation; IVC: inferior vena cava variation; IVC-DI: inferior vena cava dispensability index.

**Table 3. t03:** Comparison between groups in tidal volume of 9 mL/kg group.

	Responders (n=38)		P value	Non-Responders (n=41)		P value
	Before fluid challenge	After fluid challenge		Before fluid challenge	After fluid challenge	
HR (bpm)	65.8±11.1	61.0±9.7*	0.006	58.5±6.9^#^	56.8±8.1	0.154
MAP (mmHg)	68.5±8.1	71.1±8.3	0.116	70.0±8.4	68.8±7.2	0.337
VTI (cm)	18.1±3.0	22.4±3.4*	<0.001	20.2±2.7^#^	21.4±3.0*	<0.001
PPV (%)	15.6±4.7^¶^	7.3±2.8*	<0.001	8.4±2.8^#¶^	5.6±1.8*	<0.001
IVC max (cm)	1.8±0.2	2.1±0.3*	<0.001	1.8±0.3	2.1±0.3*	<0.001
IVC min (cm)	1.5±0.3	1.9±0.3*	<0.001	1.7±0.3^#^	2.0±0.3*	<0.001
IVC-DI (%)	17.3±9.0^¶^	5.4±3.3*	<0.001	8.6±5.3^#^	5.1±3.5*	<0.001

Data are reported as means±SD. Student's *t*-test (normal distribution) or Wilcoxon rank sum test (non-normal distribution) were used. *P<0.05 *vs* before fluid challenge; ^#^P<0.05 *vs* responders, ^¶^P<0.05 *vs* tidal volume of 6 mL/kg (see Table 2). HR: heart rate; MAP: mean arterial pressure: VTI: velocity-time integral; PPV: pulse pressure variation; IVC: inferior vena cava variation; IVC-DI: inferior vena cava dispensability index.

**Table 4.** Comparison between groups in tidal volume of 12 mL/kg group.

	Responders (n=38)		P value	Non-Responders (n=41)		P value
	Before fluid challenge	After fluid challenge		Before fluid challenge	After fluid challenge	
HR (bpm)	66.7±10.3	60.3±7.9*	<0.001	57.8±5.6^#^	57.7±7.8	0.963
MAP (mmHg)	68.5±8.4	68.6±7.5	0.984	68.5±6.7	69.4±7.6	0.453
VTI (cm)	18.1±2.9	22.2±3.3*	<0.001	20.0±2.7^#^	21.2±2.9*	<0.001
PPV (%)	20.4±6.0^¶^	9.3±3.5*	<0.001	11.0±3.5^#¶^	6.9±2.1*	<0.001
IVC max (cm)	1.8±0.2	2.1±0.3*	<0.001	1.9±0.3	2.1±0.2*	<0.001
IVC min (cm)	1.6±0.3	2.0±0.3*	<0.001	1.8±0.3^#^	2.0±0.2*	<0.001
IVC-DI (%)	14.2±8.0^¶^	5.8±5.1*	<0.001	8.5±5.9^#^	5.2±3.5*	<0.001

Data are reported as means±SD. Student's *t*-test (normal distribution) or Wilcoxon rank sum test (non-normal distribution) were used. *P<0.05 *vs* before fluid challenge; ^#^P<0.05 *vs* responders; ^¶^P<0.05 *vs* tidal volume of 9 mL/kg (see Table 3). HR: heart rate; MAP: mean arterial pressure: VTI: velocity-time integral; PPV: pulse pressure variation; IVC: inferior vena cava variation; IVC-DI: inferior vena cava dispensability index.

### Influence of fluid challenge in the R and NR groups

After fluid challenge was performed and when the 6 mL/kg TV ventilation was performed, it was observed that the HR, PPV, and IVC-DI were significantly decreased. Additionally, VTI, IVC_max_, and IVC_min_ were significantly increased in both groups. When the tidal volume increased to 9 mL/kg, it was found that the HR, PPV, and IVC-DI in the R group were significantly decreased, and the VTI, IVC_max_, and IVC_min_ values were significantly increased. In the NR group, the PPV and IVC-DI values were significantly decreased, and the IVC_max_ and IVC_min_ values were significantly increased; however, there was no significant change in HR and MAP, and such conditions were also seen when a TV=12 mL/kg ventilation was performed.

### Effect of TV on PPV and IVC-DI

For the R group, PPV and IVC-DI increased when TV increased from 6 to 9 mL/kg, while PPV increased and IVC-DI decreased when TV increased from 9 to 12 mL/kg. For the NR group, PPV increased with TV and IVC-DI did not change significantly.

### ROC curve analysis in the evaluation of the diagnostic utility of PPV and IVC-DI at different tidal volumes

**Figure 5.**
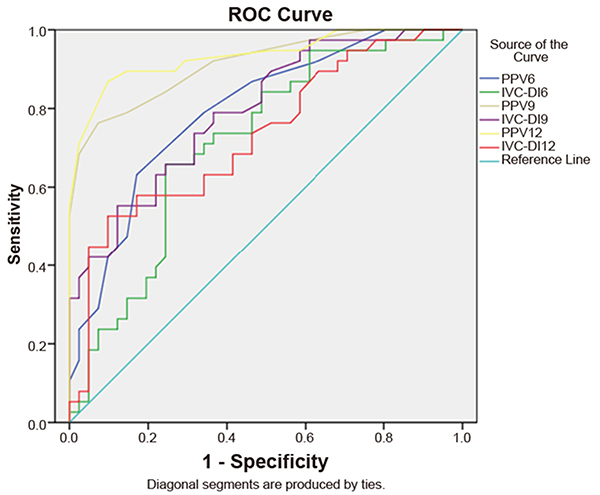
Receiver operating curve (ROC) of pulse pressure variation (PPV) and inferior vena cava dispensability index (IVC-DI) of three tidal volumes: 6, 9, and 12 mL/kg.

By plotting a receiver operating characteristic curve (Table 5 and Figure 5), when the 6 mL/kg TV ventilation was performed, we can see that the area under the ROC curve of the PPV was 0.79 (95%CI: 0.70, 0.89) and the threshold value was 8.5% (the sensitivity was 55% and the specificity was 83%). The area under the ROC curve of IVC-DI was 0.71 (95%CI: 0.60, 0.83) and the threshold value was 11.1% (the sensitivity was 68% and the specificity was 76%). When 9 mL/kg TV ventilation was performed, the area under the ROC curve (AUROC) of PPV was 0.91 (95%CI: 0.85, 0.98) and the threshold value was 12.5% (the sensitivity was 76% and the specificity was 93%). The AUROC of IVC-DI was 0.79 (95%CI: 0.70, 0.89) and the threshold value was 15.3% (the sensitivity was 55% and specificity was 88%). When 12 mL/kg TV ventilation was performed, AUROC of PPV was 0.93 (95%CI: 0.88, 0.99) and the threshold value was 15.5% (the sensitivity was 87% and the specificity was 90%). The AUROC of IVC-DI was 0.73 (95%CI: 0.62, 0.84) and the threshold value was 13.4% (the sensitivity was 53% and the specificity was 88%).

**Table 5.** Prediction of fluid responsiveness by the ROC curves of pulse pressure variation (PPV) and inferior vena cava dispensability index (IVC-DI) measured before fluid loading in different tidal volumes: 6, 9, and 12 mL/kg.

TV	AUC (95% CI)	P value	Threshold value (%)	Sensitivity (%)	Specificity (%)
6 mL/kg					
PPV	0.79 (0.70, 0.89)	<0.001	8.5	55	83
IVC-DI	0.71 (0.60, 0.83)	0.001	11.1	68	76
9 mL/kg					
PPV	0.91 (0.85, 0.98)	<0.001	12.5	76	93
IVC-DI	0.79 (0.70, 0.89)	<0.001	15.3	55	88
12 mL/kg					
PPV	0.93 (0.88, 0.99)	<0.001	15.5	87	90
IVC-DI	0.73 (0.62, 0.84)	0.001	13.4	53	88

ROC curve: receiver operating characteristic curve; AUC: area under the ROC curve; TV: tidal volume.

The results of the Delong test (Table 6) showed that Z=2.8 (P=0.005) when the AUROC of PPV9 was compared to PPV6; Z=0.99 (P=0.32) when the AUROC of PPV12 was compared to PPV9; Z=1.87 (P=0.062) when the AUROC of IVC-DI9 was compared to IVC-DI6; Z=-1.63 (P=0.103) when the AUROC of IVC-DI12 was compared to IVC-DI9; Z=-2.28 (P=0.023) when the AUROC of IVC-DI19 was compared to PPV at a TV of 9 mL/kg; and Z=-3.67 (P<0.001) when the AUROC of IVC-DI12 was compared with that of PPV at a TV of 12 mL/kg.

**Table 6.** Results of paired comparison among area under the ROC curve (AUC) by the Delong method.

	PPV6	IVCDI6	PPV9	IVCDI9	PPV12	IVCDI12
PPV6		–1.16 (0.246)	2.80 (0.005)	0 (0.996)	2.72 (0.007)	-0.87 (0.385)
IVC-DI6			3.52 (<0.001)	1.87 (0.062)	3.83 (<0.001)	0.35 (0.729)
PPV9				–2.28 (0.023)	0.99 (0.32)	–3.06 (0.002)
IVC-DI9					2.74 (0.006)	–1.63 (0.103)
PPV12						–3.67 (<0.001)

There are 6 rows and 7 columns in the matrix, which represents pairwise comparisons of the AUC areas of different indexes; the same index does not compare with itself. The z value is reported outside of the parentheses and the P value is within the parentheses. P<0.05 indicates that there was a significant difference, that is, the overall AUC area of the two indexes was different. Better performance can be seen according to the z value sign; i.e. the minus sign indicates that the column variable performed better, and vice versa.

## Discussion

Mechanical ventilation causes periodic changes in intrathoracic pressure (ITP), after which the stroke volume (SV) varies periodically. Increased ITP caused by inspiration can be transmitted at least partially to the pericardium, thereby increasing transmural pressure of the right ventricular wall. If the ventricle is in the ascending part of the Frank-Starling curve, periodic changes in ITP can cause periodic changes in ventricular preload and SV (positive volume responsiveness) (3). For dynamic indices, which include PPV and IVC-DI, changes in ITP caused by breathing must be sufficiently large to cause periodic changes in cardiac output. Among mechanically ventilated patients, TV was considered a major factor that affected the pleura, pericardial pressure, and right ventricular afterload, and increased TV can shift the Frank-Starling curve to the left (20).

This study found that PPV and IVC-DI displayed a poor diagnostic accuracy for volume responsiveness when the TV was 6 mL/kg, which is concordant with the published results of others (21,22). This might be because a low TV is insufficient to cause significant periodic changes in ITP, so that the SV is unable to change significantly, which results in PPV, and thus IVC-DI cannot be used as an index for the measurement of volume responsiveness. Moreover, excessive reliance on PPV or IVC-DI can increase the false negative rate when the TV is low (23,24).

This study noted that the diagnostic accuracy of PPV for volume responsiveness increased with commensurate increases in the TV. Myatra et al. (25) improved the diagnostic accuracy of PPV through a process referred to as “tidal volume challenge”. Similar studies (26) have also shown that the accuracy of PPV and SVV in diagnosing volume responsiveness at a TV of 10 mL/kg was significantly higher than that of a TV of 5 mL/kg. When TV increased from 6 to 10 mL/kg, the PPV increased significantly in those with volume responsiveness; however, it was not significant in those without volume responsiveness (27). Min et al. (28) observed patients that had undergone selective laparotomy under conditions of tracheal intubation and general anesthesia. When patients with a PPV in the grey interval (at 9-13%) were enrolled in the study, the PPV value increased by temporarily adjusting the TV from 8 to 12 mL/kg, thereby increasing the diagnostic value of PPV in the grey interval at a TV of 8 mL/kg.

Therefore, we might consider temporarily increasing the TV to 12 mL/kg to improve the predictive accuracy of PPV for volume responsiveness. However, if patients do not tolerate 12 mL/kg TV, then setting the TV to 9 mL/kg and the threshold value to 12.5% should translate to a diagnostic specificity of 93%, and the PPV can also accurately predict the volume responsiveness.

For IVC-DI, the diagnostic accuracy was optimal when the TV was set at 9 mL/kg. Therefore, when IVC-DI is used as a diagnostic index, there is no justification or need to increase the TV to 12 mL/kg.

This study showed that the diagnostic accuracy of PPV in diagnosing volume responsiveness is superior to that of IVC-DI when the TV values are 9 and 12 mL/kg; however, when the TV is 6 mL/kg, there is no significant difference between the PPV and IVC-DI indices. This result is somewhat concordant with that of a previously published study (29), which set the TV of patients to exceed 8 mL/kg and a PEEP of 8-10 cm H_2_O. The main conclusion is that PPV offers greater accuracy than IVC-DI in predicting volume responsiveness. Another study (30) set the TV to be 8 mL/kg, a value that also resulted in greater accuracy of PPV compared with IVC-DI in predicting volume responsiveness.

The results of this study showed that IVC-DI lacked a sufficiently high value in diagnosing volume responsiveness, which is similar to the results of the meta-analyses previously published by Long et al. (31) and Orso et al. (32). However, the meta-analysis of Si et al. (33) suggested that IVC-DI was a reliable index in diagnosing volume responsiveness under conditions where the TV ≥ 8 mL/kg and the PEEP ≤ 5 cm H_2_O. In addition, when compared with a TV < 8 mL/kg or a PEEP > 5 cm H_2_O, IVC-DI displayed greater sensitivity (0.80 *vs* 0.66; P=0.02), specificity (0.94 *vs* 0.68; P<0.001), diagnostic odds ratio (68 *vs* 4; P<0.001), and AUROC (0.88 *vs* 0.70; P<0.001).

Based on our findings and the results of other meta-analyses, we suggest that the ventilation mode had a greater impact on the diagnostic accuracy of IVC-DI, and the heterogeneity of IVC-DI was higher due to the different factors of the different meta-analyses, including the disease status of the patient, the “gold standard”, etc. Therefore, when IVC-DI is used in clinical practice, attention should be paid to the specific conditions of the patients and other indices rather than relying solely on a certain preferred or indicated index. Ultrasound has many advantages in clinical practice because of its non-invasive and convenient diagnostic value. A prior study (34) suggested that transesophageal ultrasonography for the measurement of superior vena cava variation (ΔSVC) was more accurate than PPV and IVC-DI in diagnosing volume responsiveness. Baron et al. (35) also suggested that ΔSVC was better in assessing the volume of mechanically ventilated patients. Therefore, ultrasound has great potential for its utility in clinical practice.

We found that different studies conducted different fluid challenges. In one study (8), 500 mL of a crystal or a colloid solution was used as the standard for fluid challenge. Lanspa et al. (36) injected crystals according to the metric of 10 mL/kg and the infusion time was less than 20 min. Charbonneau et al. (37) injected 6% hydroxyethyl starch according to the metric of 7 mL/kg and the infusion time was continued for 15 min. In addition, Min et al. (28) injected the crystal solution according to a metric of 6 mL/kg, which was allowed to continue for 10 min.

In 2017, a systematic review and meta-analysis (38) included 85 studies (with a total of 3601 patients) and confirmed that the fluid challenge was unaffected by the type of fluid but was instead influenced by the infusion time duration. Another study (39) showed that the volume of crystals/colloids also affected the effectiveness of the fluid challenge. Therefore, the fluid challenge in this study was 6 mL/kg of succinyl gelatin and the infusion was permitted to proceed for 10 min to balance the risk of SV change that was caused by an insufficient infusion volume, or by contrast, a volume overload that might be caused by an excessive infusion volume.

Stroke volume can be calculated by the formula SV = VTI × AA, where VTI is left ventricular outflow tract velocity-time integral and AA is aortic area. The aortic area was considered stable throughout the experiment (40). Thus, an increase or decrease in the VTI might represent an increase or a decrease in the SV, and patients that showed an increased VTI of the left ventricular outflow tract (ΔVTI) of greater than or equal to 15% following the fluid challenge were enrolled in the response group. This can also avoid bias caused by repeated measurements of AA.

Limitations of this study included the following: 1) all patients enrolled in the study were relatively healthy before surgery, thus, this study cannot be generalized to all patients; 2) ultrasound is influenced by personal experience, and the largest and smallest diameter of the inferior vena cava is differentially adjudicated by naked eye inspection, which is clearly a non-objective measurement; 3) the gold-standard for volume responsiveness is a 15% variation in cardiac output as evaluated by invasive methods (i.e., the Swan-Ganz catheter) and, in this investigation, the VTI of the left ventricular outflow tract was used as a surrogate of the cardiac output, which could have greater variability, for example, according to the transducer's angulation in sequential evaluation; 4) twelve patients were excluded because of the imaging quality in the healthy individuals that had undergone elective surgery; in real life situations, and mainly in the setting of critical care, greater attention should be paid to this technical limitation; 5) exclusion of patients that used vasoactive drugs could have inadvertently excluded patients that might present with low blood volume and volume responsiveness.

## Conclusions

The accuracy of PPV in predicting volume responsiveness can be increased by increasing the TV. The IVC-DI index can also predict volume responsiveness, at least in part, when the TV is 9 mL/kg. The diagnostic value of PPV was higher than that of IVC-DI. PPV or IVC-DI should not be used solely for fluid management, but in combination with clinical signs and other measured clinical indices. PPV or IVC-DI can only be used as a tool for evaluation and not necessarily as a target for guiding treatment management.
